# Ginger (*Zingiber officinale *Roscoe) extract can improve the levels of some trace elements and total homocysteine and prevent oxidative damage induced by ethanol in rat eye

**Published:** 2020

**Authors:** Abolfazl Akbari, Khadijeh Nasiri, Mojtaba Heydari

**Affiliations:** 1 *Department of Physiology, School of Veterinary Medicine, Shiraz University, Shiraz, Iran*; 2 *Department of Exercise Physiology, Faculty of Sport Science, University of Mazandaran, Babolsar, Iran*; 3 *Poostchi Ophthalmology Research Center, Shiraz University of Medical Sciences, Shiraz, Iran*

**Keywords:** Eye, Ethanol, Ginger, Zingiber officinale Roscoe, Oxidative stress, Homocysteine, Trace element

## Abstract

**Objective:**

Acute and chronic ethanol consumption cause oxidative stress and ginger improves suchconditions. In this study, the protective effects of ginger were studied on indices of oxidative stress, total homocysteinelevel and the level of the some of the oxidative stress-associated trace elements against toxicity induced by ethanol in rat eye.

**Materials and Methods:**

Twenty-four adult male Sprague-Dawley rats were randomly allocated into four groups and treated daily for 28 days as follows: group I: control;group II: ginger (1g/kg/day ginger extract by oral gavage); group III: ethanol (4g/kg/day ethanol by oral gavage) and group IV: ginger+ethanol. At the end of the experimental period, eye tissue sera were used for determination of different parameters. Furthermore, *in vitro* antioxidant potential and total phenol content of ginger extract were determined.

**Results:**

In ethanol group, significant changes in oxidative stress markers and levels of homocysteine and some trace elements, compared to other groups, were observed (p<0.05 for all cases). However,these parameters significantly ameliorated with pretreatment with ginger in ginger+ethanol group (p<0.05 for all cases), and had no significant differencesinthese parameters betweenginger and control group were found.

**Conclusion:**

It can be concluded that ginger extract has protective effects against toxicity induced by ethanol in the eye of male rat.

## Introduction

Over-consumption of alcohol, after smoking and hypertension, is one of the most common risk factors for disease burden in high-income countries. It is associated with chronic diseases such as cancer, and gastric, neurodegenerative and chronic ocular diseases(Crews and Nixon, 2009 ▶; Peragallo et al., 2013 ▶). Excessive consumption of alcohol is associated with an increased incidence of chronic ocular diseases such as keratitis, cataract, corneal arcus and color vision deficiencies (Hiratsuka and Li, 2001 ▶). One of the most important factors contributing to the pathogenesis of excessive consumption of alcohol and chronic ocular diseases, is oxidative stress. Oxidative stress is caused by increased production of free radicals such as reactive oxygen species (ROS) or decreased antioxidant capacity that is required to detoxify their harmful effects.The amount of ROS is controlled by antioxidant enzymessuch as superoxide dismutase (SOD), catalase andglutathione peroxidase (GPx), vitamin C and E and sulfhydryl group donors (e.g., GSH) (Akbari et al., 2016b ▶; Jelodar et al., 2018 ▶). Antioxidant enzymes and some other enzymes such as cytochrome c oxidase contain transition metal ions (e.g., Fe^2+^, Cu^2+^, Zn^2+^ and Mn^2+^) which act as integral part of their active sites to battle against free radicals (Akbari et al., 2016a ▶; Akbari et al., 2017 ▶). Therefore, abnormal homeostasis of these elements can also lead to organ dysfunction and oxidative stress. 

Another mechanism through which cell toxicity can be induced by ethanol, is its interaction with homocysteine (Hcy). Elevated homocysteine via interference with the signaling pathways, impairs intra/extracellular mechanisms. Hcy metabolism is mostly controlled by epigenetic regulations such as histone modifications, and DNA acetylation and methylation (Kamat et al., 2016 ▶). 

The eye is a sensitive organ and susceptible to oxidative damage because of its continuous exposure to environmental chemicals, radiation, and atmospheric oxygen. Oxidative stress may play a crucial role in development of ocular diseases such as uveitis, glaucoma, cataracts, age-related macular degeneration, and pseudoexfoliation syndrome (Ohia et al., 2005 ▶). Therefore, use of supplements and plant compounds to enhance the body's antioxidant system can improve the status of oxidative stress and prevent the onset of these diseases.Today, the use of medicinal plants and phytochemical products like proteins, flavonoids and phenols and other bioactive compounds, has gained popularity in preventing and treating many diseases associated with oxidative stress, including cancer, diabetes, and neurologic, reproductive and eye diseases. 

*Zingiber officinale *Roscoe (ginger) is one of the most popularmedicinal plants evaluated in multiple studies for its effect on oxidative stress. It contains numerous phytochemical compounds including zingerone, gingerdiol, zingiberene, zerumbone, gingerols and shogaols which have antioxidant properties (Akbari et al., 2017 ▶; Ilkhanizadeh et al., 2016 ▶; Tzeng et al., 2016 ▶). However, the effect of ginger on oxidative stress in eye is less investigated. The aim of this study was to evaluate the protective effect of ginger extract on indices of oxidative stress, tHcy, and some of the oxidative stress-associated trace elements in ethanol-induced toxicity in male rats.

## Materials and Methods

**Plant material and extraction**

Rhizomes of fresh ginger were purchased from aherbal shop (*Attari*) in April 2018, Shiraz, Iran. It was verified by aherbalist in Shiraz University of Medical Sciences and the voucher number PM-948 was assigned to it.

The processes of drying and powdering ginger rhizome were done atroom temperature. Then, 250 g of dried ginger powder was mixed with 70% ethanol solution in anErlenmeyer. Thesuspension was shaken using a shaker for 48 hrand then filtered through afilter paper. For evaporating the alcohol, the prepared liquid was put in an oven at40°C. The residual was the extract of ginger. By adding definite amounts of corn oil, the required dose of the extract was prepared for oral administration.

**Animals**

All stages of the experiment were performed in accordance with the "Guiding Principles for the Care and Use of Research Animals" approved by Shiraz university. Twenty-four adult male Sprague-Dawley rats (220±15g) were colony-bred (six rats per cage) in the animal room under controlled lighting (12 hr light: 12 hr darkness) and temperature (20±2ºC) and they had free access to pelleted food and tap water.

**Experimental design**

To evaluate the effects of ginger extract on toxicity induced by ethanol in eye tissue, animals were randomly allocated to four equal groups (n=6 in each group). 

Group I: Vehicle or control group which received normal saline (1 ml/day)

Group II: Ethanol group which received ethanol (4 g/kg of body weight (BW)/day)

Group III: Ginger group received ginger extract (1 g/kg of BW/day)

Group IV: Ginger-ethanol group which received ethanol (4 g/kg of BW/day) after administration of ginger (1 g/kg of BW/day).

Daily oral administration of the vehicle and extract were done for 28 days. Alcohol (ethanol 96%) was purchased from Razi Chemical Company (Tehran, Iran).

**Biochemical analysis and assessment of eye oxidative status**

After the 28th day, animalls were fasted overnight with free access to drinking water, then, sacrificed by deep anesthesia induced by ether. Eyes were removed and rinsed in ice saline. Then, homogenized manually in cold phosphated buffer (pH7.4, 0.1M) and debris were removed by centrifugation at 3000g for 10 min. The upper clear supernatants were then recovered and stored at -70ºC for assaying enzymes and total protein. To evaluate the ethanol-induced oxidative damage in eye tissue the activities of SOD, GPx and CAT and MDA level were measured. The activities of SOD and GPx were determinedby laboratory Ransel and Ransod kits (Randox Company, UK), respectively. To evaluate catalase activity ineye, we used the method described by Aebi(Aebi, 1984 ▶). The MDA levels were evaluated by a modified high-performance liquid chromatography (HPLC) method which is based on the reaction between MDA and thiobarbituric acid (TBA) to form a colored MDA-TBA adduct. 

The levels of trace elements were measured by atomic absorption spectrophotometry (Shimadzu AA-670, Kyoto, Japan) (Kubaszewski et al., 2014 ▶). Briefly, 1 ml of serum tissue and 1ml of hydrochloric acid and nitric acid solution were mixed together; then, digestion was done using 80°C water bath for 16hr. Deionized water was used for dilution of the samples to 1 ml. Dilution of 1,000 ppm certified standard solutions (Merck, Germany) was used for preparing working standard solutions of Zn, Mn, Cu, and Fe. Total homocysteine (tHcy) of the eye serum was determined by the Axis Homocysteine EIA kit.

**Statistical analysis**

Statistical analysis of data by one-way analysis of variance (ANOVA), followed by Tukey’s comparison test to compare between treated and control groups, were performed by the Statistical Package for Social Sciences (SPSS17.0). Significant statistical difference was considered at p<0.05. The results are represented as mean±standard error of mean (SEM). 

## Results

The mean values± SEM for BWand some trace elementsare presented in Tables 1 and 2, respectively. Ethanol could significantly decrease BW in the ethanol group compared to control, while preetreatment with ginger improved it in the ginger-ethanol group (p<0.05 for both cases, Table 1). The results showed that the levels of copper and manganese were not significantly different between the ethanol and control group (p<0.05, Table 2). Also, the level of iron significantly decreased in the ethanol and ethanol groups compared to the control group (p<0.05, Table 2). The results also showed that the level of zinc in ethanol group significantly increased (p< 0.001), while pretreatment with ginger significantly deccreased the level of zinc in the ginger-ethanol group compared to the control (p<0.05) and ethanol group (p<0.001, Table 2). 

**Table 1:** Changes in body weight during the study period in different groups

**Group**	**Control**	**Control+Ginger**	**Ethanol**	**Ginger+Ethanol**
**Day 0**	260.57±7.14	265.77±4.60	262.17±3.16	274.17±4.36
**Day 7**	278.73±7.28	273.48±4.67	248.34±3.37‡*	254.33±4.42‡*
**Day 14**	286.47±7.59 ‡	283.23±4.58 ‡	232.73±3.16 ‡‡*	268.33±4.2
**Day 21**	296.80±7.79 ‡	299.91±6.80 ‡‡	216.87±3.11 ‡‡*	271.89±4.21
**Day 28**	321.17±7.61 ‡‡	326.27±6.74 ‡‡‡	199.79±2.99 ‡‡‡***	279.25±3.90**###

Results are presented as mean± SEM for body weight (g). * p<0.05, **p<0.01, *** p< 0.001 compared to control group. ### p<0.001 compared to ethanol group (n=6). ‡p<0.05, ‡‡p< 0.01, ‡‡‡p<0.001 compared to prior week of study.

**Table 2:** The mean value of trace elements (µg/dl) in studied groups

		**Control**	**Ginger**	**Ethanol**	**Ginger +Ethanol **
**Eye**	**Cu**	0.13±0.04	0.1±0.01	0.183±0.014 *	0.1±0.011
	**Mn**	3.6±0.17	3.675±0.454	3.433±0.21	3.766±0.31
	**Fe**	12.66±1.47 #	13±1.4#	9±0.45 *	11.5±1.24 *#
	**Zn**	47.1±2.45	28.175±1.7 **###	115.1±11.24 ***	35.95±2.48 *###

Results are presented as mean± SEM of trace elements (µg/dl). * p<0.05, **p<0.01, *** p<0.001 compared to control group. # p<0.05, ### p<0.001 compared to ethanol group (n=6).

**Figure 1:**
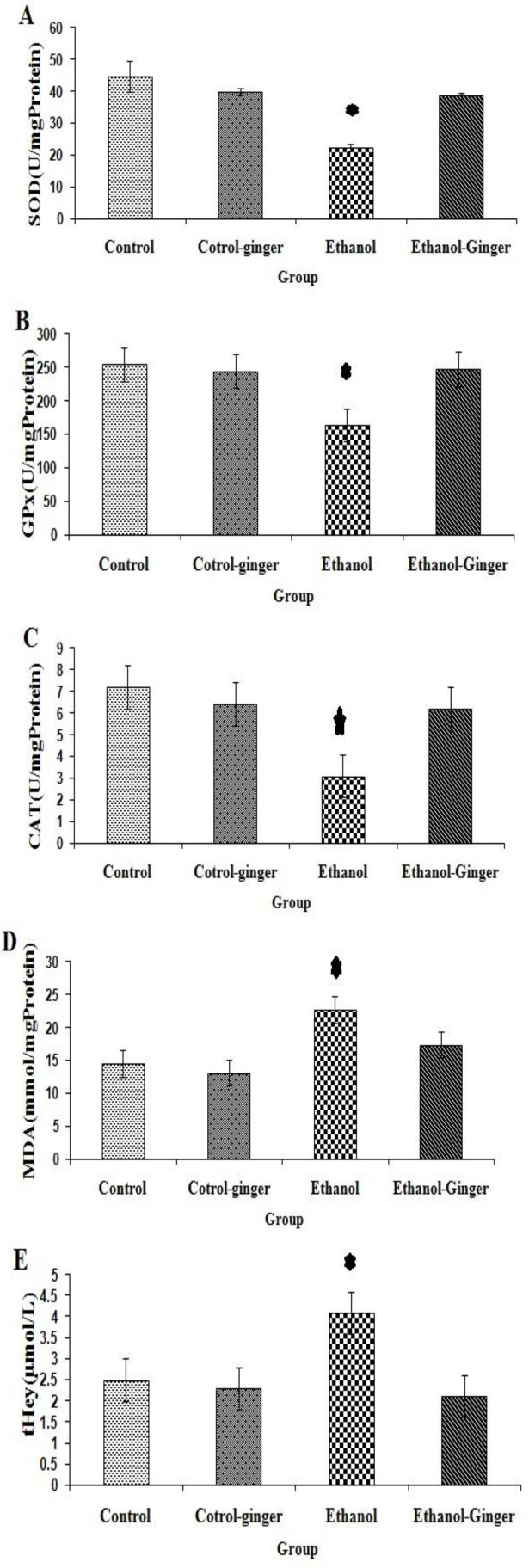
Comparison of SOD (A), GPx (B), CAT (C) and MDA (D) and tHcy (E) among studied groups (n=6). Data are expressed as mean±SEM. Asterisk indicates a significant difference among groups (p<0.05).

The protective role of ginger extract on eye oxidative status in the toxicity induced by ethanol is shown in Figure 1. The activities of SOD, GPx and CAT were significantly decreased after administration of ethanol in the ethanol group compared to the control (p<0.05 for all cases, Figure 1), while pretreatment with ginger returned their levels to normal (p<0.05 for all cases, Figures 1A-C). The level of MDA as one of the byproducts of lipid peroxidation, was measured for the evaluation of oxidative stress. In the ethanol group, MDA level increased significantly compared to other groups, while pretreatment with ginger significantly decreased it compared to the control (p<0.05 for all cases, Figure 1D). 

## Discussion

The results of this study showed that MDA and tHcy and reduce SOD,GPx and CAT in ethanol group were increased, while pretreatment with ginger could improve levels of trace elements and tHcy and oxidative stress status in ginger-ethanol group, which agrees with previous reports (Akbari et al., 2017 ▶; Albano 2006 ▶; Ilkhanizadeh et al., 2016 ▶). Studies indicated that ginger extract reduce lipid proxidation, tHcy and oxidative stress induced by ethanol (Akbari et al., 2017 ▶) and streptozotocin (STZ)-induced diabetes (Ilkhanizadeh et al., 2016 ▶). In this study, we hypothesized that disruption of homeostasis of trace elements and tHcy level have a predominant role in the induction of oxidative stress. 

However, many factors and processes are involved in oxidative stress induced by alcohol. The interaction between ethanol and trace elements at two levels are investigated. The first is the effect of ethanol on the concentration and distribution of certain trace metals such as iron, copper, selenium, manganese and zinc in the body. The second is the possible protection that exerted by some trace elements against damage-induced by ethanol. Some trace elements such as manganese, zinc, copper and selenium, can act as antioxidants to eliminate free radical (Dreosti and Pitman, 2009). 

Some studies also showed that metabolism of ethanol by increasing the NADH/NAD ratio can increase the production of hydroxyl radical (Albano, 2006 ▶). In many organs, including the eye, excessive ethanol consumption led to increased production of ROS and/or suppression of antioxidant defense mechanisms, thereby resulting in oxidative damage. It is well known that one ofthe main damages to cells was occurred by ROS via reacting to lipids, proteins and DNA, and can induce apoptosis which causes tissue injury (Rottner et al., 2011 ▶). Our results showed that ethanol causes change in levels of copper, iron, zinc and manganese and induce oxidative stress in the eye, and pretreatment with ginger could improve these parameters, which is consistent with previous reports (Akbari et al., 2017 ▶; Dreosti and Pitman, 2009). It was also reported that ethanol reduced levels of some of trace elements and induced oxidative stress in the testis, and ginger could improve these conditions (Akbari et al., 2017 ▶). The antioxidant activity of ginger comes from high level of phenolic and flavonoid compounds which act as free radical scavengers and inhibit lipid peroxidation and damage to DNA (Siddaraju and Dharmesh, 2007 ▶). Ginger contains bioactive compounds such as gingerdiol, zingerone, gingerols, shogaols and zingiberene (Zancan et al., 2002 ▶). In our study, ethanol increased MDA and tHcy concentration in the ethanol group, and in ginger-ethanol group pretreatment with ginger could restore MDA and tHcylevels to normal. Lipid peroxidation as a physiological process in membrane, plays a crucial role in tissue injury and MDA is a byproduct oflipid peroxidation. GPx as one of the antioxidant enzymes decomposes peroxides such as H_2_O_2 _and decreases lipid peroxidation by protecting cell membranes against free radicals (McCay et al., 1976 ▶). Studies have shown a positive correlation between plasma level of homocysteine and excessive consumption of ethanol (Gibson et al., 2008 ▶; Stickel et al., 2000 ▶). Chronic alcohol consumption results in hyperhomocysteinemia by decreasing plasma level of folate and vitamin B_12 _(Gibson et al., 2008 ▶), and elevated level of tHcy induces oxidative stress (Barak et al., 2001 ▶; Tyagi et al., 2005 ▶). Studies have shown that ginger via anti-inflammatory, antioxidant and antiangiogenic properties, have a protective role in streptozotocin-induced diabetes (Ilkhanizadeh et al., 2016 ▶) and ethanol-induced reproductive toxicity (Akbari et al., 2017 ▶). Ilkhanzadeh et al., (2016) ▶ indicated that ginger extract improves serum apolipoproteins A and B, tHcy and can reduce heart structural abnormalities in diabetic rats (Ilkhanizadeh et al., 2016 ▶). It is possible that ginger has effects on homocysteine synthesis. However, more studiesareneeded.

The results of the present study indicated that ethanol induces oxidative stress, changes in the level of some trace element and increases in tHcy, and ginger can improve all of these factors in rat eye. Regarding the mechanisms of regulating homocysteine synthesis and homeostasis of trace elements, and the role of the ginger extract in improving these two processes, it should be noted that ginger extract not only by its antioxidant activity but also by interference withthese two processes,was able to improve the damage caused by ethanol. Further studies usinghigher doses and longer treatment periods, could shed more light on the relevance of the current findings. It should be considered that ethanol-induced toxicity was done by gavage in this study which is obviously different from optional use of it. Self-administration methods for ginger and ethanol consumption are suggested for further investigations.
